# The contribution of FEV_1_ and airflow limitation on the intensity of dyspnea and leg effort during exercise. Insights from a real‐world cohort

**DOI:** 10.14814/phy2.14415

**Published:** 2020-04-23

**Authors:** Imran Satia, Mohammad Abdul Malik Farooqi, Ruth Cusack, Masanobu Matsuoka, Xie Yanqing, Om Kurmi, Paul M. O’Byrne, Kieran J. Killian

**Affiliations:** ^1^ Division of Respirology Department of Medicine McMaster University Hamilton ON Canada; ^2^ Firestone Institute for Respiratory Health St Joseph’s Healthcare Hamilton ON Canada; ^3^ Division of Infection, Immunity and Respiratory Medicine Manchester Academic Health Science Centre University of Manchester Manchester UK

**Keywords:** cardio‐pulmonary exercise testing, dyspnea, leg effort, physiology

## Abstract

**Rationale:**

The effort required to cycle and breathe intensify as power increases during incremental exercise. It is currently unclear how changes in FEV_1_ in the presence or absence of airflow limitation) impacts the intensity of dyspnea and leg effort. This is clinically important as the improvement in FEV_1_ is often the target for improving dyspnea.

**Objectives:**

To investigate the relationship between dyspnea (D), leg effort, power (P), and FEV_1_ with and without airflow limitation using direct psychophysical scaling performed during incremental exercise testing to symptom limited capacity.

**Methods:**

Retrospective analysis of consecutive patients over the age of 35 referred for cardio‐pulmonary exercise testing at McMaster University Medical Centre from 1988–2012.The modified Borg scale was used to measure dyspnea throughout incremental exercise testing.

**Measurements and results:**

38,788 patients were included in the analysis [Mean Age 58.6 years (*SD* ±11.8), Males 61%, BMI 28.1 kg/m^2^ (*SD* ±5.1), FEV_1_ was 2.7 L (*SD* ±0.85), 95% predicted (*SD* ±20.4), FVC 3.4 L (*SD* ± 1.0), 94% predicted (*SD* ±17.0)], and 10.9% had airflow limitation (AL, FEV_1_/FVC < 70%). In a nonlinear regression analysis, the intensity of dyspnea increased in a positively accelerating manner with power and as the FEV_1_% predicted decreased: Dyspnea = 0.06 * Power^1.03^ * FEV_1_%Pred^−0.66^(*r* = .63). The intensity of leg effort increased with power and declining quadricep strength and FEV1% predicted: Leg Effort = 0.06 * Power^1.22 ^* Quad^−0.56^*FEV_1_%Pred^−0.39^(*r* = .73). There was no independent effect of AL on dyspnea of leg effort.

**Conclusion:**

Power, quadriceps strength and FEV1 are the dominant factors contributing to dyspnea and leg effort, irrespective of the degree of airflow limitation.

## Graphical abstract

Power output, quadriceps strength, and FEV1 are the dominant factors contributing to dyspnea and leg effort, irrespective of the degree of airflow limitation.

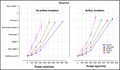

## 1 INTRODUCTION

Exercise intolerance is commonly attributed to the reduction in the capacities of the heart, lung, or neuromuscular systems. The person is limited because he cannot increase his cardiac output, ventilation, gas exchange, or achieve neuromuscular activation to meet the demands of a normal exercise capacity. Meanwhile, the subject is perceptually aware of exercise intolerance through the increase in the sense of effort required to drive the limb and respiratory muscles—leg fatigue and dyspnea.

An increased sense of effort and feeling breathless during exercise is a normal human experience that becomes alarming when the increase is disproportionate to exercise intensity or it becomes intolerable, leading to a limitation in exercise, activities of daily living and ultimately a reduction in quality of life (Waschki et al., 2011; Garcia‐Rio et al., 2012). Campbell and Howell formalized the concept of inappropriateness in the generation of dyspnea first as length tension inappropriateness which progressed to any inappropriateness such as the effort required to breathe (Campbell and Howell, 1963). The role of the brain in processing sensory inputs and providing motor output to both limb and respiratory muscles and their perceptual consequencesis also central to the issue of exercise intolerance, but seldom quantified.

Classical direct psychophysics quantifies the relationship between the magnitude of a stimulus and the intensity of its perceptual response (Stevens, 1958). The direct physical stimulus involved in activation of the limb and respiratory muscle is the central motor command to the respective muscles. Psychophysiological testing is thus an extension of classical psychophysics. In the context of exercise, the intensity of dyspnea and leg fatigue can be directly measured relative to power and the factors contributing to the variability across subjects that can be identified and quantified using a direct psychophysical method.

The goal of the limb muscles is to generate power, while the goal of the respiratory muscles is to generate sufficient ventilation to meet the gas exchange demands of the exercising muscle. Similarly, the heart must also generate sufficient power to support the blood flow to the exercising muscle and the pulmonary circulation. Thus, the heart, lungs, and circulation must work together to achieve gas exchange in the lungs and convect oxygen delivery to the muscles and carbon dioxide excretion. Failure of such convection leads to rapid fatigue due to the inability to regenerate ATP via oxidative metabolism.

During incremental exercise, there is a finite limit to the maximum power that can be generated by the limb muscles. For the respiratory system, the finite limit is defined by the maximum breathing capacity (MBC), which approximates to 40× the FEV1, which is sufficiently accurate and useful for clinical purposes. The objective of this study was to investigate the contribution of FEV1 to the effort required to cycle (leg effort) and breathe (dyspnea), relative to power output and quadriceps strength.

## 2 METHODS

The intensity of dyspnea was recorded from rest to symptom limiting exercise capacity on a cycle ergometer relative to the power generated and FEV_1_ in both absolute terms and expressed as percentage of predicted normal values based on the contributions of height, age, and sex in the normal population. The rationale was to use the intensity of dyspneaand leg effort as the dependent variable while pursuing the independent factors contributing to this intensity with attention to power, FEV_1_, and the presence or absence of airflow limitation (AL). All subjects provided informed consent to perform cardio‐pulmonary exercise testing since 1988 and provided consent for the anonymized data to be used for clinical audit and research purposes.

### 2.1 Study design

This was a retrospective analysis of all patients over the age of 35 referred for cardio‐pulmonary exercise testing at McMaster University Medical Centre from 1988–2012.

### 2.2 Subjects

This analysis included all patients referred for cardio‐pulmonary exercise testing over the age of 35 with no exclusions reflecting real world experience in a clinical setting.Variation in FEV_1_ due to height, age, and gender was adjusted by expressing the value as % of predicted normal values. The reference values were generated based on local data FEV1 = [0.9 + (0.13 in males)] * Height^2.40^ * (1 − (0.008 * Age > 35) * (1−(0.006 * Age < 20), which means FEV1 increases in a positively accelerating manner with height, is proportionately higher in males than females (at any given height), is unchanged between the ages of 20–35, and then declines by a fractional proportion per year after 35 and increases with age up to 20. Our normal standards have been reviewed and updated since the 1970s. Our current normal population contains 17,000 normal subjects from Hamilton, Ontario, Canada.

The subject population included patients with cardiovascular and congenital heart disease, patients with obstructive and nonobstructive pulmonary disease, patients screened prior to rehabilitation programs in the elderly and normal subjects.

### 2.3 Study procedures

Prior to exercise, muscle strength, spirometry, gas transfer capacity of the lung for carbon monoxide (TLCO/DLCO) and arterialized capillary blood gases (CBG) were measured. During exercise, oxygen uptake, carbon dioxide output, respiratory quotient (RQ), cardiovascular factors heart rate (HR), blood pressure (BP), electrocardiogram (ECG); respiratory factors ventilation(VE), tidal volume (VT), respiratory rate (RR), end tidal and mixed expired carbon dioxide (PetCO2, PeCO2) and oxygen saturation (SaO_2_) were measured each minute following incremental exercise to symptom limited capacity. The intensity of leg and breathing effort and chest pain were measured by matching their perceived intensity to quantitative semantics attached to numbers on the modified Borg Scale (0–10) at every increment of power. The power outputs (PO) started at 0 and increased by 100 kpm/min in a stepwise manner up to maximum capacity. The final power output was defined as the maximum power output (MPO).

### 2.4 Statistical analysis

Single and multiple linear, nonlinear regression, and MANOVA were used. Demographic data are shown as mean and standard deviation (*SD*). The intensity of dyspnea and leg effort were the dependent variables. The independent variables were power, FEV_1_(L) and % predicted, quad strength. Age, sex, and height were added as independent variables in the multi‐variate model. All data are illustrated using the mean and 95% confidence intervals.

## 3 RESULTS

### 3.1 Subject demographics, physiology, and exercise data

In total, 38,788 consecutive patients underwent an incremental exercise test to limiting capacity between 1988 and 2012 (Table 1). Sixty one percent were male; the mean age was 58.6 years (*SD* ±11.8), height 1.69 m (*SD* ±0.01), BMI 28.1 kg/m^2^ (*SD* ±5.1). The mean FEV_1_ was 2.7 L (*SD* ±0.85), 95% predicted (*SD* ±20.4); FVC 3.4 L (*SD* ± 1.0), 94%predicted (*SD* ±17.0); FEV1/FVC was 79% (*SD* ±8.9), 11.8% demonstrated chronic airflow limitation (CAL, FEV_1_/FVC < 70%). The mean maximum power output (MPO) achieved was 758 kpm/min (*SD* ± 328), 83% predicted (*SD* ±25.3).

**TABLE 1:** Baseline demographics, pulmonary function, muscle strength and exercise testing

Variable	*N*	Mean	*SD*
Demographics			
Age	38,788	58.59	11.8
Height (m)	38,787	1.69	0.1
BMI (kg/m^2^)	38,785	28.12	5.1
Baseline physiology			
FEV1 (L)	38,742	2.68	0.8
FEV1%pred	38,737	95.08	20.4
FVC (L)	38,727	3.40	1.0
FVC %pred	36,223	93.82	17.0
FEV1/FVC%	38,724	78.77	8.9
DLCO (ml/minHg/min)	36,291	21.96	6.6
DLCO%pred	33,799	92.22	20.6
Muscle strength			
MIP (cmH_2_0)	38,525	74.39	30.4
MEP (cmH_2_0)	38,552	106.62	37.5
Quads strength (kg)	35,007	45.73	19.7
Exercise test			
MPO (kpm/min)	38,750	758.27	327.6
MPO%pr	38,619	83.00	25.3
VO_2_ max (L/min)	38,275	1.60	0.7
VE at MPO (L)	38,737	57.69	23.4
HR at rest	38,588	76.88	14.8
HR at MPO	38,641	134.62	26.1
SaO_2_ at rest	38,311	96.64	2.0
SaO_2_ at MPO	38,556	95.68	3.0

Note

Abbreviations: BMI, Body mass index; FEV1, forced expiratory volume in 1 s; FVC, forced vital capacity; HR, heart rate; MEP, mouth expiratory pressure; MIP, mouth inspiratory pressure; MPO, maximum power output; oxygen saturations; SaO_2_; TLCO, total lung diffusion capacity to carbon monoxide; VE, ventilation; VO_2_, rate of oxygen consumption.

### 3.2 Factors contributing to maximum power output

**FIGURE 1:**
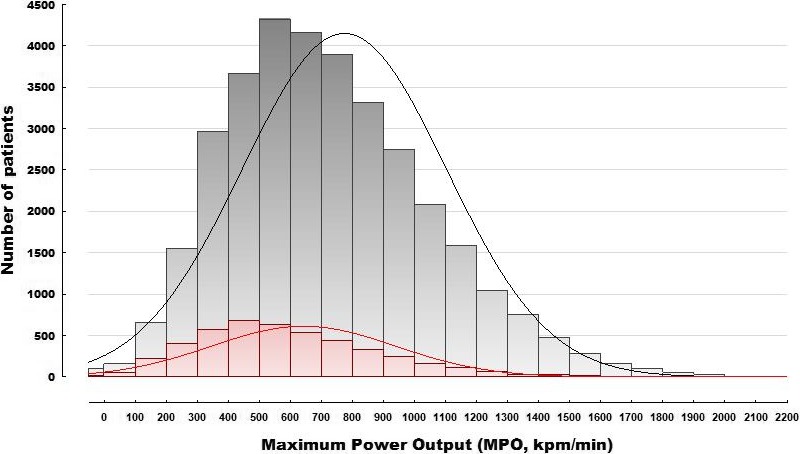
Distribution of maximum power in all patients with and without airflow limitation. Red histogram bars and line depicts patients with airflow limitation (FEV1/FVC < 70%)

There was a significant reduction in the absolute maximum power output (MPO) achieved and the % predicted in patients with airflow limitation. The mean MPO was 630 kpm/min (*SD* ±310), 68% predicted (*SD* ±26) with airflow limitation compared to 748 kpm/min(*SD* ±353), 78% predicted (*SD* ±28) in those withoutairflow limitation(*p* < .0001)(Figure 1). Multiple additive linear regression analysis showed that the MPO increased with quadriceps and FEV1 and declined with age and the contribution of airflow limitation, age, height, and sex were minor (Table 2).The intercept was not significantly different from 0.

**TABLE 2:** Factors contributing to the maximum power output

	Std Beta	*β*	*p*‐value
Quadricep strength (kg)	.44	7.09	.000000
FEV_1_ (L)	.36	135.55	.000000
Age	−.14	−2.58	.000000
Airflow limitation (FEV1/FVC < 0.7)	.02	26.06	.000000
Height (m)	.04	136.86	.000000
Gender (*M* = 1)	.06	41.19	.000000
Weight (kg)	−.02	−.33	.000002

Note

Multiple linear regression analysis performed. r = 0.8191, r^2^ = 0.67, SEE = 191.

### 3.3 Contribution of power, FEV1% predicted, and airflow limitation to dyspnea

**FIGURE 2:**
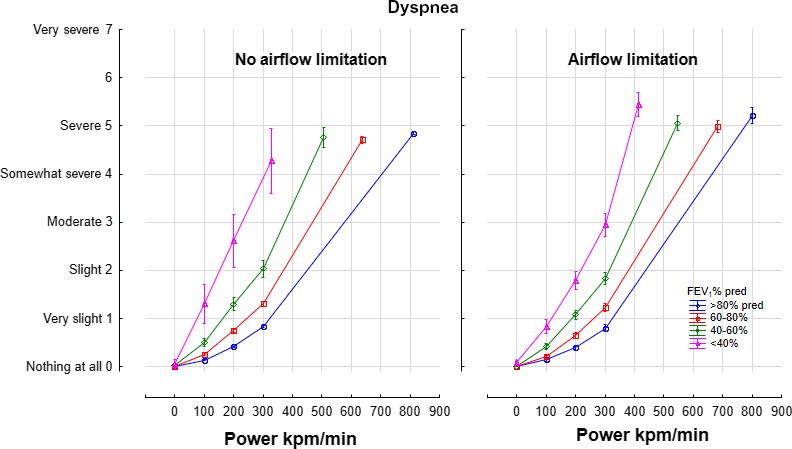
Intensity of dyspnea on a mBorg scale relative to power, FEV1% predicted and the presence of absence of airflow limitation. Means ± 95% Confidence Intervals

The intensity of dyspnea increased with power and decreasing FEV1, andthe contribution of airflow limitation, age, height, and sex were minor (Table 3). In a non‐linear and interactive model, the intensity of dyspnea increased with power and declining FEV_1_% predicted in an interactive manner:Dyspnea = 0.06 * Power^1.03^ * FEV_1_%Pred^−0.66^ (*r* = .63). The presence or absence of airflow limitation did not contribute significantly. Power outputs of 0,100, 200, 300 and maximum power output (kpm/min) are shown in the presence or absence of airflow limitation (Figure 2).

**TABLE 3:** Factors contributing to dyspnea

	Std Beta	*β*	*p*‐value
Intercept		1.39	.000000
Power (kpm/min)	.70	.0015	.000000
FEV_1_ (L)	−.16	−.41	.000000
Airflow limitation (FEV1/FVC < 0.7)	.01	.08	.000000
Age	.03	.00	.000000
Height (m)	−.01	−.22	.000007
Gender (*M* = 1)	−.07	−.32	.000000
Weight (kg)	−.01	−.00	.000000

Note

Multiple linear regression analysis performed. *r* = .6519, *r* ^2^ = .42, SEE = 1.69.

### 3.4 Contribution of power, quadricep strength, FEV1% predicted, and airflow limitation to leg fatigue

**FIGURE 3:**
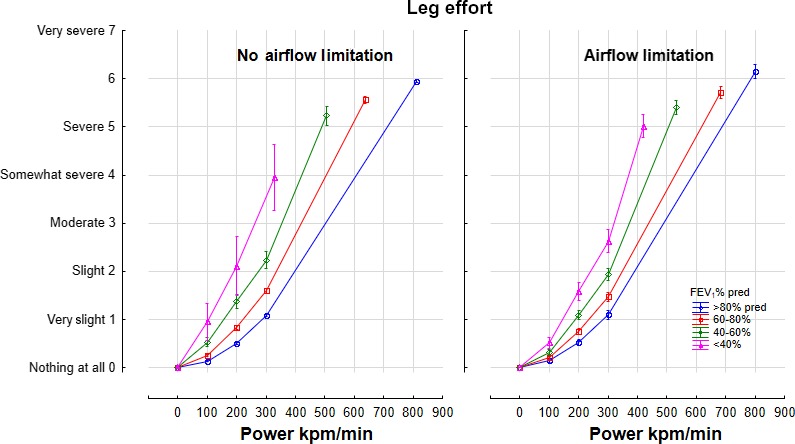
Intensity of leg effort on a mBorg scale relative to power, FEV1% predicted and the presence of absence of airflow limitation. Means ± 95% Confidence Intervals

The intensity of leg effort increased with power and decreasing quadriceps strength and FEV1.The contribution of airflow limitation, age, height, and sex were minor (Table 4). In a nonlinear and interactive model, the intensity of dyspnea increased with power and declining quadricep strength and FEV_1_% predicted in an interactive manner: Leg Effort = 0.06 * Power^1.22 ^* Quad^−0.56 ^* FEV_1_%Pred^−0.39^ (*r* = .73). The presence or absence of airflow limitation did not contribute significantly. Power outputs of 0,100, 200, 300, and maximum power output (kpm/min) are shown in the presence or absence of airflow limitation (Figure 3).

**TABLE 4:** Factors contributing to leg fatigue

	Std Beta	*β*	*p*‐value
Intercept		1.41	.000000
Power (kpm/min)	.78	.01	.000000
Quadricep strength (kg)	−.18	−.02	.000000
FEV_1_ (L)	−.10	−.30	.000000
Airflow limitation (FEV1/FVC < 0.7)	.01	.07	.000000
Age	−.02	−.00	.000000
Height (m)	.00	.07	.188835
Gender (*M* = 1)	−.02	−.11	.000000
Weight (kg)	.02	.00	.000000

Note

Multiple linear regression analysis performed. *r* = .7217, *r* ^2^ = .52, SEE = 1.76.

## 4 DISCUSSION

In this study, the perceived intensity of dyspnea and leg effort during incremental exercise was explored in a quantitative manner across a very broad range of patients in a real‐world clinical setting. The intensity of dyspnea and leg effort positively accelerates with power and reaches its maximum intensity at the maximum power output. The rate of acceleration of dyspnea increases with power output and declining FEV1% predicted. While the intensity of leg effort also increases with power output, and declines as quadricep strength and FEV1% predicted declines. Importantly, the presence or absence of airflow limitation, defined physiology as an FEV1/FVC ratio of <0.7 had no additional impact.

By concentrating on increasing power to symptom limiting capacity,the complex inter‐relationships between the composite function of all the unit processes in sustaining oxidative respiration have been accounted for (Jones & Killian, 2000). These are undoubtedly complex involving multiple organ systems; the lungs, heart, muscles, afferent and efferent nerves, acid‐base balance, and brain. The objective of the exercising subject is to match ventilation to the metabolic demand (VO_2_ and VCO_2_), in addition to acid base balance in the muscle. As local muscle acidosis increases, neural respiratory drive must increase ventilation to prevent acidemia, and it is this increased sense of effort in ventilation that subjects describe as dyspnea. A declining FEV1 implies a reduction in the capacity to breathe using maximum inspiratory and expiratory effort. Likewise, quadricep strength reflects the maximum capacity to generate power by the legs. With this physiological view, the presence or absence of airflow limitation fails to contribute to the capacity to exercise, and the effort required to breathe or cycle at any given power.

The FEV1 was introduced in the 1950’s by Tiffeneau and Pinelli and resulted in a revolution (Tiffeneau and Pinelli, 1948). It was quickly adopted as a measurement of ventilatory capacity. Without validation 35–40 times the FEV1 was taken to approximate the MBC with enough accuracy for clinical utility. By the late 1950s it was recognized that the inspiratory and expiratory flow pattern matched that seen with a maximal inspiratory and expiratory maneuver(Kennedy, 1953). During incremental exercise to capacity the maximum tidal volume approaches 60% of the vital capacity. By calculating the minimum time required to move 60% of the vital capacity using the techniques described, 40 times the FEV1 is sufficiently close to MBC with a Pearson *r* approaching .9. Hence ventilation during exercise expressed relative to MBC approximates respiratory effort in a simple uncomplicated manner but structured on classical mechanics. In this context, it becomes more clear why if a subject has evidence of obstructive airways diseases (FEV1/FVC < 0.7), that the capacity to breathe is solely expressed by the FEV_1_.

The pertinent outcome in patients with pulmonary disease is improvement in the capacity to exercise and reduction in the intensity of dyspnea experienced. When evaluating and managing patients with dyspnea, FEV1 is the most commonly evaluated measurement. What does this measurement actually mean to the patient? The equations presented in this manuscript allow for the evaluation of any given change in FEV1 to the capacity to exercise and the intensity of dyspnea at any given power. From regression analysis in Table 2, MPO improves by 135 kpm/min for every Liter change in FEV1. Improvements in clinical trials of bronchodilator therapy in breathless patients with chronic obstructive pulmonary disease (COPD) amount to approximately 200 ml; this is associated with an estimated increase in MPO by 27 kpm/min. Similarly, a 10% absolute improvement in FEV1 will provide an estimated improvement in dyspnea by 13% using the non‐linear equation described in Figure 2. Clearly, the intensity of dyspnea is multifactorial and only in part related to FEV1.

One might argue that the most pertinent endpoint for any therapeutic intervention should be the capacity to exercise and the intensity of dyspnea experienced. Studies have assessed improvements in transition dyspnea index (TDI), endurance capacity at a constant work rate, and inspiratory capacity (IC). A large pair‐wise network meta‐analysis of 8 published and unpublished studies including 6 randomized controlled trials in 1632 patients with moderate to severe COPD showed LABA/LAMA therapy to improve exercise endurance by 60 s in a constant work rate exercise protocol and improve IC by 229 ml compared with placebo (Calzetta et al., 2017). Bronchodilatation reduces the end expiratory lung volume (EELV), breathing starts at a lower point in the pressure‐volume curve, requiring less ventilatory drive and reduced intensity of dyspnea (O'Donnell, Revill, and Webb 2001; O'Donnell, Voduc, Fitzpatrick, & Webb, 2004; O'Donnell, Fluge, et al., 2004). While this is true, one should not ignore the fact that hyperinflation allows a much greater capacity to breathe because ventilatory capacity is greatest where the combination of maximum inspiratory and expiratory flow rates are maximum. Even normal subjects hyperinflate to reach their maximum breathing capacity. Whatever the mechanisms, these studies demonstrated modest clinically improvement with a modest prolongation in endurance time. O’Donnell and colleagues reported a modest reduction in the modified Borg scale during a constant work rate in the intensity of dyspnea (0.740 and 0.693 Borg units) with tiotropium/oladaterol at 2.5/5 and 5/5 μg compared with placebo (O'Donnell et al., 2017). Maltais and colleagues demonstrated greater improvements in the modified Borg Scale units of 1.33 with LABA/LAMA and −0.97 with LAMA alone, but without a placebo arm using a shuttle walk test allowing a measurement of exercise capacity (CSST) (Maltais et al., 2019).

Historical reporting of subjects using indirect psychophysical techniques have also been used. The transition dyspnea index (TDI) has been used as a measure of dyspnea during day‐to‐day activities, and a pooled analysis of longitudinal data using indacaterol over 52 weeks demonstrated that a 100 ml improvement in FEV_1_ yields a 0.46 unit increase in TDI, where 1 unit is considered the minimum clinically improvement difference (MCID) (Jones et al., 2011). Subsequent studies with dual bronchodilator therapy did show a greater probability of achieving an improvement >1 unit in the TDI score versus patients on monotherapy, but mean differences did not achieve the 1 unit threshold (Bateman et al., 2013; Miravitlles, Urrutia, Mathioudakis, & Ancochea, 2017; Singh, Worsley, Zhu, Hardaker, & Church, 2015). All indirect techniques are based on memory and do not reflect the less variable direct assessment of perceived symptom intensity under directly applied stimulus conditions that we have applied in this study.

A distinction also has to be made between the beneficial changes in bronchodilatation resulting in improved performance during an exercise test and actual day‐to‐day physical activity. A recent study evaluated the benefits of a self‐management behavior modification (SMBM) program in addition to dual bronchodilators and exercise training. The study demonstrated increased physical activity by 10–11 min per day, but exercise and inhaler therapy did not significantly increase physical activity(Troosters et al., 2018). This was surprising given that the study showed evidence of increase exercise endurance testing and resting inspiratory capacity, with an associated reduction in the intensity of dyspnea during exercise. The discordance between expiratory flow rates and real‐world physical activity highlights the importance of the mind in performing physical activity. Motivation, confidence, the desire to ambulate and support independence of thought and action modify the subjective perception of symptoms.

The strength of this study was the study population taken from a real‐life setting over a 25 years and a direct psychophysical test was performed. For all indirect techniques, patient memory and bias are inherent limitations and can be avoided by direct psychophysical testing. When a physician encounters a patient reporting dyspnea, the pursuit of the disease condition must be broadened to include responder bias. This is where anxiety or mood disorders are associated with an exaggerated response and a stoical status with a reduced response.

There are some limitations worth nothing. First, this is a retrospective, observational, and population‐based study performed in one center. It is unclear whether similar results would be demonstrated in an interventional study within an individual over time. Second, the broad range of patients studied in this analysis may be considered inappropriate in that the patient diagnosis is not defined. However, by not defining the patients, a much broader view of dyspnea and its relationship with power can be achieved. Third, over prolonged periods of time, temporal adaptation results in a marked reduction in symptom intensity at rest and is of limited value.

## 5 CONCLUSION

The intensity of dyspnea and leg effort are important components in the ability to exercise and are the final factor limiting the capacity to continue exercising. We have demonstrated that power output, FEV1 and quadricep strength are all important contributors to the intensity of symptoms and the capacity to exercise. The presence of or absence of airflow limitation defined by FEV1/FVC ratio <0.7 has no significant independent influence.

## CONFLICT OF INTEREST

IS reports grant from BMA James Trust Award, grants from North West Lung Centre, Merck, personal fees from Educational Talks for GPs, sponsorship to attend conference meetings, outside the submitted work; MAM, RC, MM, XY, OK, KK all have no disclosures. POB reports personal fees from oversight committee for LABA safety study, consulting fees from Astrazenca, GSK, Merck, Boehringer, grants from Astrazeneca, Genentech, outside the submitted work.

## AUTHOR’S CONTRIBUTIONS

IS, POB, and KK contributed to concept and design. IS and KK contributed to statistical analysis and modeling. All authors reviewed the manuscript and approved the final draft.

## Data Availability

Raw data were generated at McMaster University (Medical Diagnostics Unit). Derived data supporting the findings of this study are available from the corresponding author [I.S] on request. The data are not publicly available due to their containing information that could compromise the privacy of participants.
